# The Evolutionary Fate of Mitochondrial Aminoacyl-tRNA Synthetases in Amitochondrial Organisms

**DOI:** 10.1007/s00239-021-10019-z

**Published:** 2021-07-12

**Authors:** Gabor L. Igloi

**Affiliations:** Institute of Biology III, University of Freiburg, Schaenzlestr. 1, 79104 Freiburg, Germany

**Keywords:** Aminoacyl-tRNA synthetase, Identity elements, Amitochondrial, Mitosome, Protozoa, Arginyl-tRNA

## Abstract

During the endosymbiotic evolution of mitochondria, the genes for aminoacyl-tRNA synthetases were transferred to the ancestral nucleus. A further reduction of mitochondrial function resulted in mitochondrion-related organisms (MRO) with a loss of the organelle genome. The fate of the now redundant ancestral mitochondrial aminoacyl-tRNA synthetase genes is uncertain. The derived protein sequence for arginyl-tRNA synthetase from thirty mitosomal organisms have been classified as originating from the ancestral nuclear or mitochondrial gene and compared to the identity element at position 20 of the cognate tRNA that distinguishes the two enzyme forms. The evolutionary choice between loss and retention of the ancestral mitochondrial gene for arginyl-tRNA synthetase reflects the coevolution of arginyl-tRNA synthetase and tRNA identity elements.

**Supplementary Information:**

The online version contains supplementary material available at 10.1007/s00239-021-10019-z.

## Introduction

The evolution of eukaryotes was dictated by the endosymbiotic acquisition of an α-proteobacterial cell and its subsequent development to the aerobic energy-generating mitochondrial organelle. Over time, the genome of the engulfed prokaryote became reduced with many of the gene products required for mitochondrial metabolism becoming-encoded by genes transferred to the nucleus whereas some were retained to permit organelle-specific protein synthesis. Among the genes transferred from the endosymbiont to the nucleus were those of the family of aminoacyl-tRNA synthetases whose products are then imported to participate in mitochondrial protein biosynthesis. However, their subsequent presence in modern eukaryote cells is very variable (Igloi 2020b). The mitochondrial gene of arginyl-tRNA synthetase, for example, has either been retained in the nucleus (e.g., in Metazoans), eliminated such that the cytoplasmic form is imported into the organelle (e.g., in higher plants), or has itself replaced the ancestral nuclear gene (e.g., in Fungi; Karlberg et al. 2000; Brindefalk et al. 2007; Furukawa et al. 2017)). This evolutionary variation within cells performing oxidative phosphorylation also raises the question as to the fate of mitochondrial aminoacyl-tRNA synthetases, in general, and of arginyl-tRNA synthetase, in particular, in the genome of modern amitochondrial, anaerobic eukaryotes. As is common usage, the product from the ancestral nuclear gene is referred to as the “cytoplasmic” enzyme, whereas the translation product of the nuclear-encoded mitochondrial gene is termed “mitochondrial” enzyme.

Arginyl-tRNA synthetase is responsible for the accurate attachment of L-arginine to its cognate tRNA in the first step leading to ribosomal protein chain extension. The specific recognition of tRNA by its cognate aminoacyl-tRNA synthetase is determined by identity elements positioned within the tRNA structure. Identity elements have been well-studied after being initially proposed (Loftfield et al. 1968) and experimentally verified (Schulman and Pelka 1989; McClain 1993). In the case of arginyl-tRNA synthetase detailed kinetic investigation of yeast transcript variants (Sissler et al. 1996) and a study of yeast tRNA mutants conferring lethality in vivo (Geslain et al. 2003b) showed that C35 (the 2^nd^ base of the anticodon) was the major identity element with minor contributions from U/G36. In the hamster cytoplasmic system (Guigou and Mirande 2005) and in plants (Aldinger et al. 2012) additionally position A20 in the D-loop emerged as an essential determinant. A20 is also a major site of recognition in bacteria (Tamura et al. 1992; McClain 1993; Shimada et al. 2001a) and its tight binding to the enzyme has been confirmed by crystallography (Stephen et al. 2018) but has been ruled out as such in yeast, both by transcript studies (Liu et al. 1999) and by the viability of mutants in vivo (Geslain et al. 2003b).

Evolutionary aspects of identity element divergence between bacteria and yeast have been discussed (Nameki et al. 1995). The phylogenetic reconstruction of yeast nuclear genes showed that a small group of aminoacyl-tRNA synthetases, including arginyl-tRNA synthetase, occurred by gene duplication from the mitochondrial lineage followed by loss of the homologue from the cytoplasmic lineage (Karlberg et al. 2000).A similar phylogenetic clustering of the nuclear-encoded yeast cytoplasmic and mitochondrial arginyl-tRNA synthetases has been noted (Brindefalk et al. 2007; Furukawa et al. 2017). It is apparent that the yeast enzyme and hence its mode of tRNA recognition originates from an ancient gene replacement of the nuclear gene by that of the mitochondrial gene. As a result of a similar gene replacement, the single valyl-tRNA synthetase gene in *Homo sapiens* provides a product with both cytosolic and mitochondrial functions and is of mitochondrial origin (Brown and Doolittle 1995; Wolf et al. 1999). A duplication of mitochondrial threonyl-tRNA synthetase and of alanyl-tRNA synthetase in eukaryotes with displacement of the ancestral eukaryotic form has also been proposed (Doolittle and Handy 1998; Wolf et al. 1999; Chihade et al. 2000).

Mammalian mitochondrial tRNA recognition has been reviewed in detail (Salinas-Giegé et al. 2015; Krahn et al. 2020) and that of arginyl-tRNA synthetase examined in insects (Igloi and Leisinger 2014) but in view of the bizarre tRNA structures of metazoan tRNAs (with reduced or missing D stem-loops, making the location of position 20 uncertain) this can not necessarily be extrapolated to conventional tRNA structures in protists. The recognition of mitochondrial tRNAs in non-metazoans having canonical secondary structures appears to follow the “universal identity rules” (Salinas-Giegé et al. 2015). tRNA recognition in protists has, with few exceptions (Cela et al. 2018) not been subjected to extensive study. However, an indication that the identity rules as far as tRNA^Arg^ is concerned may extend to non-eumetazoans can be gained from the fact that in a heterologous system, the (non-metazoan, mitochondrial-like) yeast arginyl-tRNA synthetase, where position 20 is not a recognition element, is able to aminoacylate both poriferan mitochondrial tRNA^Arg^ isoacceptors possessing either A20 and U20 (Igloi and Leisinger 2014). It is tempting, then to use the presence or absence of the tRNA-A20 nucleotide in eukaryotes possessing canonical tRNA structures, to distinguish between the recognition by cytoplasmic and mitochondrial enzymes. Thus, organisms featuring solely tRNA^Arg^A20 could survive with either the cytoplasmic or mitochondrial enzyme whereas others with non-A20 or a mixture including A20 would of necessity require the less stringent recognition by the mitochondrial enzyme which might lead to the evolutionary elimination the primordial nuclear gene.

Sequence analysis and comparisons of arginyl-tRNA synthetase has revealed a clear and characteristic distinction between the cytoplasmic and the mitochondrial forms of the enzyme. In the N-terminal region the cytoplasmic enzyme has a conserved domain recognizable by a GDYQ-like motif (whose function remains to be established). This motif is also found in some bacteria, including *E. coli* which relies on the tRNA-A20 recognition element and may be a relic of the ancestral prokaryotic host of the proto-endosymbiont. It is absent from the prokaryotic suborder Cystobacterineae from which the mitochondrial arginyl-tRNA synthetase has been derived (Igloi 2020a).The mitochondrial enzyme, on the other hand, has, when aligned with the cytoplasmic version a barely discernible GDYQ-like motif but a five-amino acid deletion followed by an MSTR-like sequence nearer the C terminus (referred to here as Δ5MSTR). This region is part of the conserved signature sequence motif KMSK which is a characteristic of the class I aminoacyl-tRNA synthetases, in general (but, exceptionally, is degenerate in arginyl-tRNA synthetases) (Sekine et al. 2001). It constitutes a part of the catalytic site in three dimensions. Although these distinctive features have been confirmed in hundreds of species from numerous phyla (Igloi 2020b), one is aware of the pitfalls in making global generalizations since it is likely that evolutionary niches with diverging characteristics will be found.

Exceptionally, in some protists gene loss in mitochondria has proceeded to completion giving rise to mitochondrion-related organelles (MRO) lacking all DNA and, hence, mitochondrial protein expression (Makiuchi and Nozaki 2014). Having eliminated mitochondrial protein synthesis, the question concerning the fate of the now redundant ancestral mitochondrial aminoacyl-tRNA synthetase genes arises. In order to trace the ancestral source of the arginyl-tRNA synthetase gene in amitochondrial organisms, the arginyl-tRNA synthetase encoded by their genomes has been examined and classified as originating from the lost mitochondrial genome or as being the retained nuclear species. Since the coevolving cognate tRNA must respond in terms of identity elements to changes in the nuclear-encoded arginyl-tRNA synthetase, a comparison of the nature of the identity element at position 20 of the corresponding nuclear tRNA^Arg^, shows a trend relating the tRNA identity element to the subcellular source of the arginyl-tRNA synthetase gene.

## Methods

Protein sequences were manually extracted from whole genome shotgun (WGS) and transcriptome (TSA) databases following TBLASTN analysis using sequences from closely related organisms or phyla. Protein sequences were derived from genomic sequence hits using the FGENESH + protein-based gene prediction algorithm (Solovyev 2008). This relies on the accessibility of genome-specific parameters which are lacking for most protists so that the data from closely related organisms was used. Considering the extent of N-terminal variability between arginyl-tRNA synthetases from different species (Igloi 2019), this section of the predicted protein sequence must be treated with caution until confirmation from the corresponding transcriptome sequence becomes available. The alternative genetic code for ciliates was used for translating the Trepomonas (Keeling and Doolittle 1997) and Streblomastix (Keeling and Leander 2003) genes. Alignments were performed with the MAFFT server (Katoh and Standley 2013) using the E-INS-I parameters and depicted in GENEDOCv2.7 (Nicholas and Nicholas 1997) with similarity groups enabled.

tRNA sequences were mined manually from the corresponding genome or transcriptome collections by BLASTN and/or with tRNAscan-SE (Chan and Lowe 2019).

## Results

**Fig. 1:**
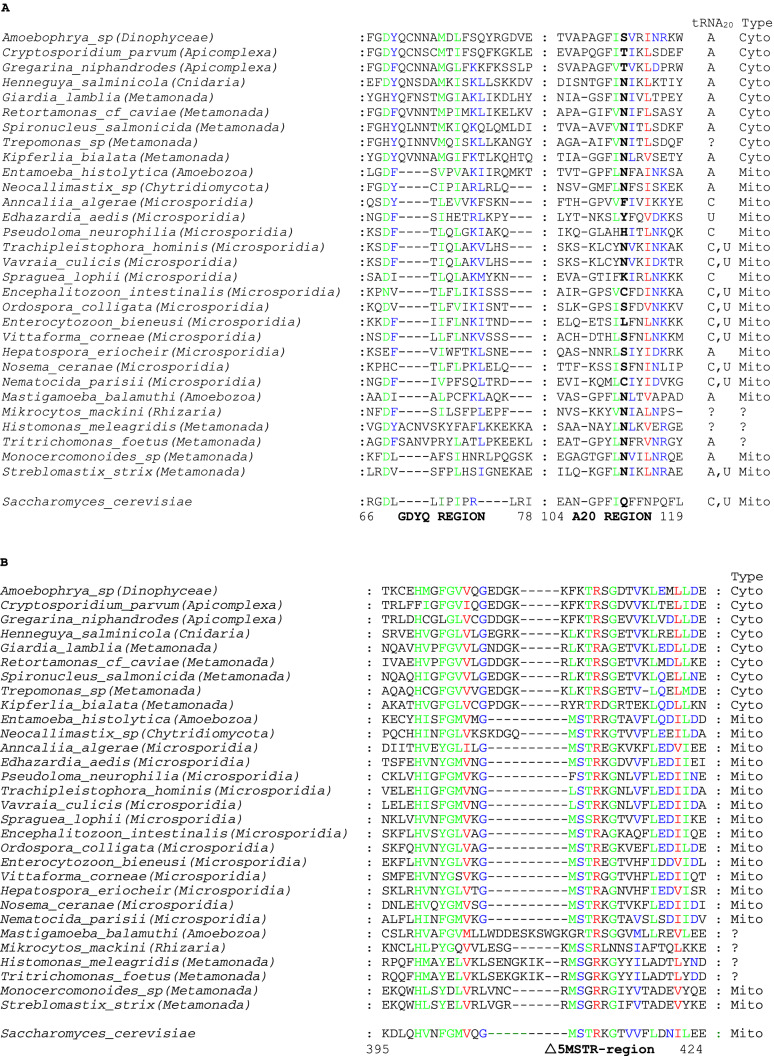
Multiple sequence alignments of arginyl-tRNA synthetase protein sequences derived from genomic or transcriptome data. **A**. Alignment of the GDYQ-like signature region and of the proposed tRNA nucleotide A20 binding-region (bold) within the arginyl-tRNA synthetase of mitosomal organisms. Columns to the right give the nucleotide at position 20 of the cognate tRNA and the ancestral source of the enzyme. **B**. Alignment of the MSTR-like signature region within the arginyl-tRNA synthetase of mitosomal organisms. Column to the right gives ancestral source of the enzyme. For orientation, the sequence of the cytoplasmic *S. cerevisiae* protein and its numbering is appended. Shading colours denote 100% identity (red), 80–100% identity (green) and 60–80% identity (blue). Cyto and Mito indicates cytoplasmic and mitochondrial characteristics of the enzyme, respectively (Color figure online)

From 6 phylogenetic clades, 30 organisms that are known to possess the MRO (mitosomes or hydrogenosomes) and for which BLAST searches have been able to identify either annotated or similarity-defined arginyl-tRNA synthetase genes have been compiled. Their putative translation products were classified (Table 1; Online Resource 1) as being of mitochondrial or of nuclear origin on the basis of the sequence motifs described above and aligned (Fig. 1). Following a highly variable N-terminus, the most obvious N-terminal region of similarity tends to commence close to the typical GDYQ-like domain in products from the nuclear-encoded gene. The genomes of the organisms were searched for as many different tRNA^Arg^ isoacceptors as possible and the nature of the base at N20 was examined and summarised (Table 1).

**Table 1:** Summary of mitosomal organisms analysed for their arginyl-tRNA synthetase (ArgRS)

Clade or Higher Classification	Phylum	Class/Order	Species	Predicted ArgRS Type	tRNA N20
Alveolata	Apicomplexa	Eucoccidiorida	*Cryptosporidium parvum*	Cyto	A
			*Gregarina niphandrodes*	Cyto	A
	Dinophyceae	Syndiniales	*Amoebophrya ceratii*	Cyto	A
Amoebozoa		Archamoeba	*Entamoeba histolytica*	Mito	A
			Mastigamoeba balamuthi*	?	A
Eumetazoa	Cnidaria	Bivalvulida	*Henneguya salminicola*	Cyto	A, C^*^
Fungi	Chytridiomycota	Neocallimastigales	*Neocallimastix sp*	Mito	A
	Microsporidia		*Anncaliia algerae*	Mito	C
			*Edhazardia aedis*	Mito	U
			*Encephalitozoon intestinalis*	Mito	C, U
			*Enterocytozoon bieneusi*	Mito	C, U
			*Hepatospora eriocheir*	Mito	A
			*Nematocida parisii*	Mito	C, U
			*Nosema ceranae*	Mito	C, U
			*Ordospora colligata*	Mito	C, U
			*Pseudoloma_neurophilia*	Mito	C
			*Spraguea lophii*	Mito	C
			*Trachipleistophora hominis*	Mito	C, U
			*Vavraia culicis*	Mito	C, U
			*Vittaforma corneae*	Mito	C, U
Metamonada	Fornicata	Diplomonadida	*Giardia lamblia*	Cyto	A
			*Kipferlia bialata*	Cyto	A
			*Retortamonas cf. caviae*	Cyto	A
			*Spironucleus salmonicida*	Cyto	A
			*Trepomonas sp*	Cyto	?
	Preaxostyla	Oxymonad	*Monocercomonoides sp*	Mito	A
			*Streblomastix strix*	Mito	A, U
	Parabasalia	Tritrichomonadida	*Tritrichomonas foetus*	Mito?	A
			*Histomonas meleagridis*	Mito?	?
Rhizaria	Endomyxa	Ascetosporea	*Mikrocytos mackini*	Mito?	?

Columns to the right indicate the predicted ancestral type and the identity element at position 20 of the cognate tRNA^Arg^

^*^For *Henneguya salminicola*, and Mastigamoeba, see text. Uncertain predictions or missing tRNA data are shown by “?” Cyto = cytoplamsic, Mito = mitochondrial

### Amoebozoa

Amoebozoa are a major taxonomic group most of whose members possess a mitochondrial genome and consequently perform mitochondrial protein biosynthesis. Their nuclear genome encodes both cytoplasmic and mitochondrial forms of the enzyme (Igloi 2020b). Nevertheless, certain Archamoeba are amitochondrial. The parasitic Entamoeba species are remarkable with their mitochondria being reduced to mitosomes and lacking organelle protein biosynthesis. In its multiple protein alignment with other amoebozoa the Archamoeba form a striking outgroup (Igloi 2019). The sequence of the protein quite clearly indicates a mitochondrial origin. It not only has a merely rudimentary cytoplasmic N-terminal GDYQ-motif but an explicit mitochondrial Δ5MSTR signature (Fig. 1), which is characteristic for fungal and all 492 metazoan mitochondrial arginyl-tRNA synthetases. The Δ5MSTR region is confirmed by EST data (Acc. No. CX094868). The gene for this protein was evidently transferred to the nucleus prior to loss of the endosymbiotic genome. A transfer of mitochondrial genes to the nucleus of *E. histolytica* and other amitochondrial organisms has been previously documented (Clark and Roger 1995; Hashimoto et al. 1998; Tovar et al. 1999; Bakatselou et al. 2003; Luque et al. 2008). The mitochondrial enzyme will recognize all four nuclear tRNA^Arg^ isoacceptors that possess A20 (Table 1). The tRNA genes are more likely to have originated from the ancestral endosymbiotic host since all available sequences of aerobic amoebozoa mitochondrial-encoded tRNA^Arg^ possess U20.

In contrast, the free-living Archamoeba *Mastigamoeba balamuthi* is also amitochondrial (Gill et al. 2007). Its arginyl-tRNA synthetase is exceptional as it cannot be unambiguously classified according to the criteria used for hundreds of other eukaryotic species (Igloi 2020b). It possesses a barely recognizable GDYQ-like domain but its Δ5MSTR signature which aligns poorly with other cytoplasmic Amoebozoa arginyl-RNA synthetases (not shown) is highly atypical but with some similarity to the cytoplasmic form. The structure of this region is, nevertheless, confirmed by EST data (Acc.No. EC698286). Furthermore, all three nuclear-encoded tRNA^Arg^ isoacceptors have nucleotide A20 and would be recognized by both enzyme types.

### Apicomplexa

Within the Apicomplexans, Cryptosporidium (Hikosaka et al. 2013) and Gregarina (Toso and Omoto 2007) possess only mitosomes. Their genomes carry the cytoplasmic form of the arginyl-tRNA synthetase and correspondingly, all tRNA^Arg^ carry the A20 identity element.

 Of the other classes within the Alveolata, Dinophyceae, are represented by the endoparasite *Amoebophrya ceratii,* which has lost its mitochondrial genome while retaining the organelle itself (John et al. 2019). It uses the cytoplasmic arginyl-tRNA synthetase in conjunction with the A20 identity element.

### Eumetazoa

The Cnidarian *Henneguya salminicola* is the sole representative of a eumetazoan anaerobe to date that survives in its host without requiring a mitochondrion (Yahalomi et al. 2020). Its cytoplasmic arginyl-tRNA synthetase has been detected in both WGS and TSA databases and the genomic data provides sequences corresponding to single copies of four tRNA^Arg^ isoacceptors. Interestingly, tRNA^Arg^_UCG_ is reported with C20 (Acc.No. SGJC01004408). Without intending to cast unfounded doubt on this sequence, one should point out that it has close similarity to the genomic sequence from its host *Oncorhynchus kisutch* (Acc. No. MPKV02000020) and the related *Salmo salar* (Acc. No. AGKD04837854), both with C20, but, remarkably, the equivalent *S. salar* TSA (Acc. No. GBRB01035513) is identical to the genomic sequence except for having A20. This may be indicative of a rare but not unknown C-to-A editing event (Smith et al. 1997; Paul et al. 2020) that would make it accessible for recognition by the cytoplasmic enzyme.

### Fungi

Fungi, in general, are characterised by having replaced the ancestral nucleus-encoded version of arginyl-tRNA synthetase gene by the mitochondrial gene (Igloi 2020b). This trend is retained in the mitosomal *Neocallimastix sp* (Chytridiomycota) (Van Der Giezen et al. 1997) but, as pointed out previously for Chytridiomycota in general (Igloi 2020b), with a reduced deletion at the Δ5MSTR region. The group of related Microsporidia have lost the mitochondrial genome but have retained its organelle-derived arginyl-tRNA synthetase (Fig. 1A and B). The genomic tRNA isoacceptors of all 13 species listed, have either A, C or U at position 20 requiring recognition by the less discriminating mitochondrial enzyme.

### Metamonada

All three phyla within the Metamonada harbour mitosomal organisms. The arginyl-tRNA synthetases, of Fornicata, represented here by five genera are, according to the sequence criteria, clearly of nuclear origin.

Within the Oxymonad order, the product of the *argS* in Streblomastix (Keeling and Leander 2003) has lost the GDYQ-feature, whereas the mitochondrial Δ5MSTR signature is evident, albeit with a reduced deletion. It is dependent on the recognition of the tRNA^Arg^U20-encoded by the organism (Table 1) so that one can classify it as mitochondrial with some confidence. Alignment with the sequence from the other available Oxymonad in the compilation, the mitosomal *Monocercomonoides sp* (Karnkowska et al. 2016) shows high similarity (with 56% identity overall, not shown) and although the tRNA isoacceptors all possess A20, the enzyme has been classed as originating from the ancestral mitochondrion. Similarly, the two closely related amitochondrial candidates from the Tritrichomonadida order, *Tritrichomonas foetus* (Lindmark and Muller 1973) and *Histomonas meleagridis* (Mielewczik et al. 2008) are highly similar in their protein alignments; 46% identity (not shown). The GDYQ-domain is barely discernible and the MSTR-feature resembles more closely that of the mitochondrial enzyme, although again the characteristic five-amino acid deletion is missing. In Tritrichomonas the cognate genomic tRNA^Arg^ has the A20 identity element which is recognizable by both enzyme types. No tRNA^Arg^ information from Histomonas is currently available.

### Rhizaria

For the single example whose mitochondrion has undergone loss of it genome, *Mikrocytos mackini* (Burki et al. 2013)*,* the multiple sequence alignment resembles in its signature regions the mitochondrial enzyme. Unfortunately, its transcriptome has, as yet, failed to reveal any tRNA^Arg^ candidate sequences.

## Discussion

Endosymbiosis led to the transfer of most of the genes from the chromosome of the proto-symbiont prokaryote to the chromosome of the host proto-eukaryote. Genes required for the metabolism of modern-day mitochondria were compulsorily retained within the nuclear genome with the translation products being imported to the organelle. Redundant house-keeping genes that had nuclear equivalents performing identical functions could be eliminated. Such genes included those for the family of aminoacyl-tRNA synthetases. This however meant an evolutionary choice between retaining the host or the symbiont gene. In the case of eumetazoans, whose non-canonical, truncated mitochondrial tRNAs (Watanabe 2010) are not substrates for the cytoplasmic enzymes (Kumazawa et al. 1991), the genes for both mitochondrial and cytoplasmic forms of the enzymes had of necessity to be retained. However, in protists, having canonical mitochondrial tRNA structures (Gray et al. 1998) retention of the mitochondrial form is optional, depending on availability of matching tRNA identity elements (and the requirement for a mitochondrial signal peptide). For amitochondrial organisms, on the other hand, for which all components of the protein synthesising machinery are located in the cytoplasm, it is not immediately obvious which evolutionary option regarding elimination of aminoacyl-tRNA synthetase genes has been taken. One possibility, investigated here, is that differences in identity element recognition might be a key aspect in matching the retained enzyme with the genomic tRNA isoacceptors.

**Fig. 2:**
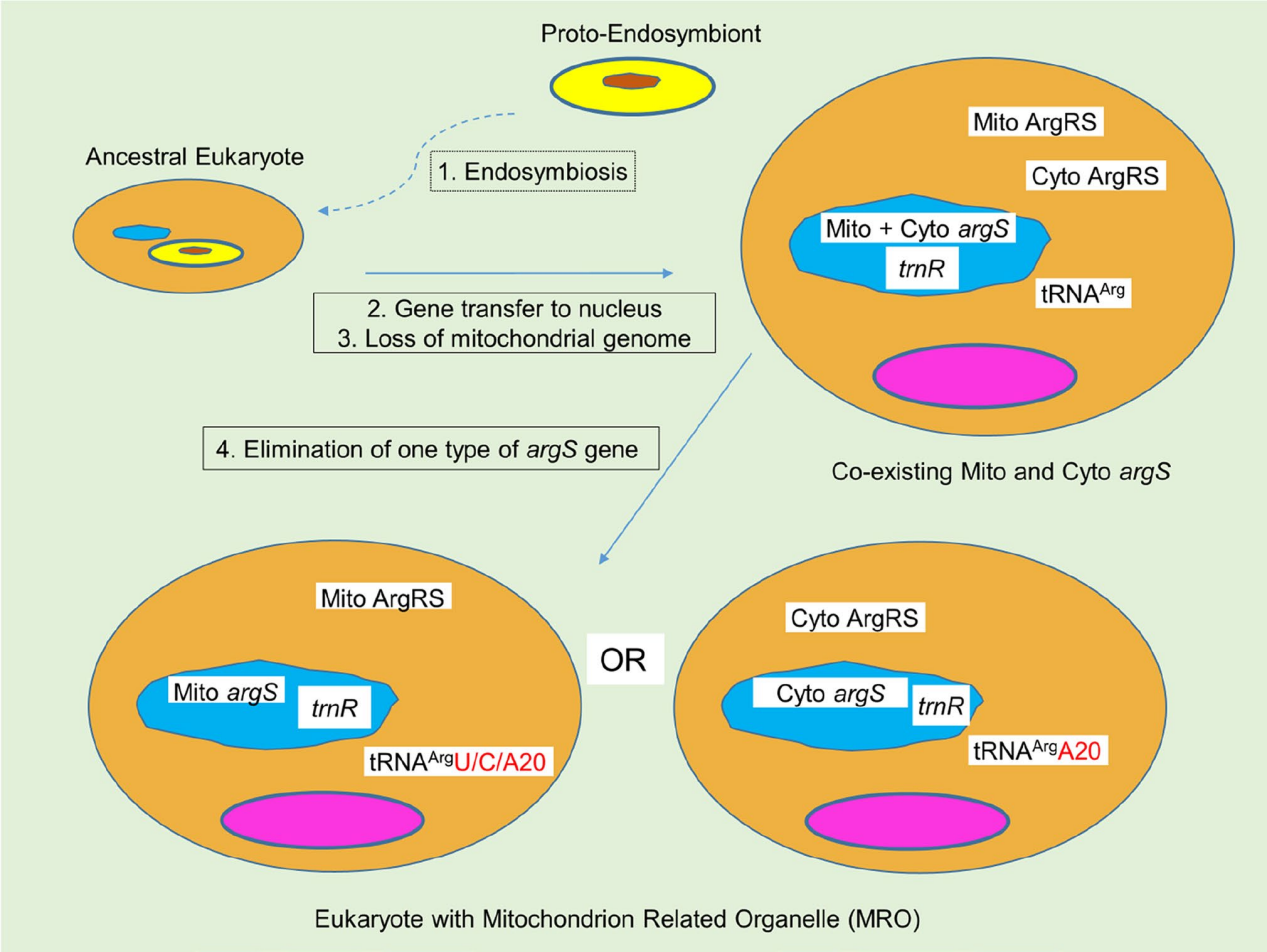
Model for the evolutionary option between retention and loss of the ancestral mitochondrial arginyl-tRNA synthetase provided by the proto-endosymbiont (yellow oval, with brown genome) in mitosomal organisms. The residual mitosome is shown in purple; the nuclear genome in blue. The genes for arginyl-tRNA synthetase and tRNA^Arg^ are symbolized by *argS* and *trnR*, respectively. Their gene products are Mito ArgRS, Cyto ArgRS and tRNA^Arg^ with the identity element given in red (Color figure online)

In this model (Fig. 2), following endosymbiosis, gene transfer from the proto-endosymbiont to the host nucleus and loss of the mitochondrial genome, an evolutionary pathway leads to the elimination of either one of the redundant arginyl-tRNA synthetases. The choice may involve the nature of the identity element present in the expressed tRNA isoacceptors. For this enzyme position 20 in the tRNA is the critical difference between recognition by the two types of enzyme. Whereas the mitochondrial form is indifferent to the nature of the nucleotide at position 20, the nuclear-encoded cytoplasmic synthetase is strictly dependent on the presence of A20. Consequently, the cognate tRNA with U20 or C20 (G20 has not been documented in any tRNA^Arg^, to date) demands the aminoacylation activity of the mitochondrial enzyme, permitting the elimination of the nuclear form. This is exemplified by the genome of Microsporidia. If all tRNA^Arg^ isoacceptors of an organism have A20 then they will be recognized by both forms of the enzyme and may retain either the mitochondrial form, as in Entameoba, or the cytoplasmic version, as in Apicomplexa. Remarkable in this respect is that Metamonada seem to be divided into two groups; the Fornicata, retaining the cytoplasmic enzyme and Preaxostyla/ Parabasalia that have opted for the mitochondrial form.

The interaction of the A20 identity element with arginyl-tRNA synthetase of bacteria has been examined in crystallographic detail (Shimada et al. 2001a). It was proposed that the amino acid equivalent to Q111 in yeast is replaced by N in bacteria. This is involved in A20 recognition and appeared to be phylogenetically invariant in the arginyl-tRNA synthetases from all organisms which possessed A20 in their tRNA^Arg^. The accumulation of eukaryotic data in the intervening years has shown that N at this position, although common, is by no means universal in non-metazoans with T being the preferred replacement (Igloi 2019). In the case of the mitosomal organisms an examination of the aligned sequences with the presence or absence of A20 in the cognate tRNA reveals a degree of agreement with the requirement for asparagine for tRNA-A20 interaction in both cytoplasmic and mitochondrial enzymes. The Microsporidia enzymes binding to U20 or C20 possess a wide variety of amino acids at the critical position (Fig. 1). In yeast, mutagenesis of F109 and/or Q111 to alanine had no effect on the viability of the organism or on the enzyme activity in vitro (Geslain et al. 2003a). Hence the mitochondrial enzymes are insensitive at this position as far as U20 or C20 are concerned but, with the exception of Hepatospora, possess N when tRNA-A20 needs to be bound (Fig. 1). On the other hand, the major deviation from the combination of N in the protein and A20 in tRNA is seen in Apicomplexa and Dinophyceae with T or S replacing N. (Fig. 1). However, the structural study of bacterial arginyl-tRNA synthetase showed that mutagenic replacement of N by D, for instance, had little influence on the kinetics of the enzyme in vitro (Shimada et al. 2001a) so that other amino acids, such as S or T might also be able to provide the H-bond that enables the critical interaction.

The nature of aminoacyl-tRNA synthetases in mitosomal organisms has been addressed in studies applied to the evolution of these enzymes with some data emerging, notably, in the case of Giardia. For alanyl-tRNA synthetase the sequences from all mitochondrion-containing eukaryotes, are of the mitochondrial type (Chang et al. 2012). However, the *alaRS* gene in the amitochondriate protists *Giardia lamblia* and *Trichomonas vaginalis* appears to have evolved from archaeal (nuclear) origin (Chihade et al. 2000; Bunjun et al. 2000; Chang et al. 2012). Similarly, Giardia tryptophanyl-tRNA synthetase is more similar to its cytoplasmic form (Arakaki et al. 2010). On the other hand, the analysis of Giardia valyl-tRNA synthetase clearly indicates that lateral gene transfer occurred between the ancestral genomes of extant mitochondria and the ancestral nuclear genome of Giardia (Brown and Doolittle 1995; Hashimoto et al. 1998) leading to the replacement of the ancestral nuclear gene.

The decision of which will be lost must then depend on additional criteria which provide an evolutionary advantage. Viability can, evidently, be achieved by either pathway*. E. histolytica* with tRNA^Arg^A20 has clearly retained the mitochondrial form whereas numerous other organisms with the same identity element from a variety of phyla have opted for the cytoplasmic version. One may speculate that with a more flexible, less stringent set of identity elements, the mitochondrial enzyme can react more easily to changes/mutations in its substrate. On the other hand, relaxed identity elements can lead to mutational tRNA misrecognition (Igloi and Leisinger 2014) and translational error propagation. Indeed there are indications that mitochondrial protein synthesis is less stringent in terms of accuracy (Shimada et al. 2001b; Lue and Kelley 2005; Roy et al. 2005) and a case in point is the high frequency of mistranslation that has been reported in microsporidia (Melnikov et al. 2018). The multiple identity elements required by the cytoplasmic enzyme (at least A20 and C35 Shimada et al. 2001a; Guigou and Mirande 2005; Aldinger et al. 2012), while making it more dependent on the tRNA structure may thereby enhance the accuracy of translation.

The concept discussed here relies on the major assumption that tRNA identity elements that are required for accurate recognition by the corresponding aminoacyl-tRNA synthetases have been co-evolutionarily conserved since the endosymbiotic acquisition of mitochondria. When it comes to evolution, it is tempting but hazardous to make global generalizations; the samples numbers in question cannot be representative of all evolutionary niches. However, the assumption regarding co-evolutionary conservation is generally admitted. Indeed, the statement that a “general trend is the global conservation throughout evolution of the identity set for a given amino acid” (Bonnefond et al. 2005) has not been questioned. Furthermore, the current evidence suggests that mitochondrial tRNAs with canonical structure (e.g., those found in plants and fungi) seem to follow the so-called universal identity rules (Salinas-Giegé et al. 2015). Additionally, the model presented here relies on the tRNA identity elements differing between cytoplasmic and mitochondrial pairs. The need for this distinction in eukaryotes performing mitochondrial protein synthesis has been elucidated in detail (Lovato et al. 2001) for alanyl-tRNA synthetase but is generally applicable.

Within the limited sample size of amitochondrial organisms, the evolutionary choice between loss and retention of the ancestral mitochondrial gene for arginyl-tRNA synthetase reflects the coevolution of arginyl-tRNA synthetase and tRNA identity elements. How this model responds to the additional complexity provided by the mitochondrial and plastid genomes and their corresponding aminoacyl-tRNA synthetase/tRNA pairs in aerobic non-metazoans remains to be established.

## Supplementary Information

Below is the link to the electronic supplementary material.

Supplementary file1 (PDF 174 KB)
