# Supplementary material for: The Evolutionary Fate of Mitochondrial Aminoacyl-tRNA Synthetases in Amitochondrial Organisms
Source: J Mol Evol. 2021 Jul 12;89(7):484–93. doi: 10.1007/s00239-021-10019-z (PMC8318970; doi:10.1007/s00239-021-10019-z)
Supplement: Supplementary file 1 — Supplementary file1 (PDF 174 KB) [file 239_2021_10019_MOESM1_ESM.pdf]

Supplementary Table 1

Accession numbers of database entries containing sequences corresponding to arginyl-tRNA synthetase and/or to a tRNAArg isoacceptor of the given species. Numbers linked with "+" denote non-overlapping contigs. tRNA data for Trachipleistophora hominis was obtained from https://fungi.ensembl.org/Trachipleistophora_hominis_gca_000316135/Info/Index. Shading in blue and brown denote the cytoplasmic and mitochondrial forms of the enzyme, repectively. Brown lettering on a blue background are enzyme forms with an uncertain ancestry.

Species	ArgRS Type			tRNA Isoacceptor (Acc. No.) (Examples from specified species or from within genus)				
	Cyto		Mito	Anticodon				
	Acc. No. Genomic	Acc.No.TSA	Acc. No. Genomic or TSA	CCU	ACG	UCU	CCG	UCG
Cryptosporidium parvum	LKCK01000001			JTAI01000007	NW_002196570	NW_002196570		
Gregarina niphandrodes	AFNH02000827			KK211487		KK211552	KK211471	KK211548
Amoebophrya ceratii	RXOD01003835					GGWB01017496	GGWB01031572	
Entamoeba histolytica			DS571166	BK005652	BK005651	BK005654	BDEQ01000001	
Mastigamoeba balamuthi			CACRSC010000031	CBKX010022044	CBKX010021216	CBKX010004149		
Henneguya salminicola	SGJC01001155	GHBP01002348<br>GHBP01003244			SGJC01006590	SGJC01007727	SGJC01004211	SGJC01004408
Neocallimastix sp			MCOG01000382			MCOG01000124		MCOG01000069
Anncaliia algerae			AOMW02000387		KK358326	KK358361		KK365146
Edhazardia aedis			AFBI03000038	AFBI03000012	AFBI03000062			AFBI03000025
Encephalitozoon intestinalis			NC_014422		ECI_CH06	ECI_CH08		ECI_CH07
Enterocytozoon bieneusi			ABGB01000023			MNPJ01000020		MNPJ01000020
Hepatospora eriocheir			KU695715	LVKB01000017	LVKB01000254			
Nematocida parisii			NW_013524647		JH605025	JH605024		JH605027
Nosema ceranae			NW_003314016		ACOL01000054	ACOL01000168		ACOL01000248
Ordospora colligata			NW_014575399		JOKQ01000007	JOKQ01000009		JOKQ01000008
Pseudoloma_neurophilia			LGUB01000003			LGUB01000003		LGUB01000001
Spraguea lophii			ATCN01000719		ATCN01000571	ATCN01000565		
Trachipleistophora hominis			ANCC01000664	scaffold00300	scaffold00042	scaffold00062		scaffold00103
Vavraia culicis			AEUG01000092		GL877475	GL877433		GL877408
Vittaforma corneae			AEYK01000027		JH370132	JH370141		JH370155
Giardia lamblia	ACVC01000232				VDLU01000005	WBSJ01000017		CM016925
Kipferlia bialata	BDIP01002268 +<br>BDIP01003232					BDIP01004586		
Retortamonas cf. caviae		GIVK01044614			GIVK01003715	GIVK01045086		
Spironucleus salmonicida	AUWU01000436			KI546057	KI546167	KI545975		KI545953
Trepomonas sp	GDID01002015							
Monocercomonoides sp	LSRY01000098	GEEL01003120		LSRY01000172	LSRY01000960		LSRY01000319	

Streblomastix strix			SNRW01013772		SNRW01020003	SNRW01009543	SNRW01000192	SNRW01033452
Tritrichomonas foetus		CAJHQR010000613		MLAK01000578	MLAK01000871		MLAK01001149	MLAK01001393
Histomonas meleagridis			GAAM01000401 + GAAM01003507					
Mikrocytos mackini			GAHX01001006					
